# Depressive symptoms and perceived burdens related to being a student: Survey in three European countries

**DOI:** 10.1186/1745-0179-4-19

**Published:** 2008-07-03

**Authors:** Rafael T Mikolajczyk, Annette E Maxwell, Vihra Naydenova, Sabine Meier, Walid El Ansari

**Affiliations:** 1 Department of Public Health Medicine, School of Public Health, University of Bielefeld, Germany; 2 School of Public Health and Jonsson Comprehensive Cancer Center, University of California, Los Angeles, USA; 3 Faculty of Sport, Health & Social Care, University of Gloucestershire, Gloucester, UK; 4 Division of Cancer Prevention and Control Research, University of California, Los Angeles 650 Charles Young Dr. South, Los Angeles, CA 90095-6900, USA

## Abstract

**Background:**

Despite a high prevalence of depressive symptoms among university students, few studies have examined how this mental health problem is associated with perceived stress and perceived burdens related to being a student.

**Methods:**

We conducted a cross-sectional study of 2,103 first year students from one western (Germany), one central (Poland), and one south-eastern European country (Bulgaria). The self-administered questionnaires included the modified Beck Depression Inventory and Cohen's Perceived Stress Scale. A 13 item scale measured perceived burdens related to being a student with four subscales: "Course work", "Relationships", "Isolation", and "Future".

**Results:**

Depressive symptoms were highly prevalent in all three countries (M-BDI ≥35: 34% in Poland, 39% in Bulgaria, and 23% in Germany). Students felt more burdened by course work and bad job prospects ("Future") than by relationship problems or by feelings of isolation. The perceived burdens subscales "Future", "Relationship" and "Isolation" remained associated with depressive symptoms after adjusting for perceived stress, which displayed a strong association with depressive symptoms. The association between perceived stress and depressive symptoms differed by gender. These findings were similar in all three countries.

**Conclusion:**

Perceived burdens related to studying are positively associated with higher depression scores among students, not only by mediation through perceived stress but also directly. While the strong association between perceived stress and depressive symptoms suggests the need for interventions that improve stress management, perceived burdens should also be addressed.

## Background

Depressive symptoms have been identified as a health problem among college or university students in many countries [1-7]. Depressive symptoms or depressive moods are different from a clinical diagnosis of depression, and are usually assessed using self-reported frequencies of feeling sad, nervous, hopeless, worthless, etc. Moderate depressive symptoms have been reported in as many as 43% of students in Central-Eastern European countries and 31% of students in Western Europe [8]. A recent review reported mild or moderate symptoms of depression in up to 25% of medical student populations in the U.S. and Canada [9]. In a representative study conducted in 1997, more than a quarter of German students reported that a mental health problem was affecting their ability to study, with a higher fraction among females than males (30% vs. 24%, respectively) [10]. Among mental health problems, depressive mood was the third most common, reported by 18% of respondents.

Despite the high prevalence of depressive symptoms in student populations, little is known about their association with specific aspects of student life, especially in the initial phase of being a student when many changes occur and adaptation processes take place [11]. The potentially relevant aspects of student life are not only learning related, but may include social interactions with peers, a possible change of residence – away from family and friends, and a changing financial situation. The extent to which these changes are perceived to be burdens by students may depend on their coping mechanisms, social support, and ability to adapt [4,8,11]. These perceived burdens could independently influence mental health or could be conceptualised as stressors. In general, stress occurs when there are demands on an individual that exceed his or her coping capabilities [12]. Perceived stress and depressive symptoms are usually strongly associated [13]. However, it is not clear whether the effect of perceived burdens on depression can be fully explained by perceived stress or whether feeling burdened is independently associated with depressive symptoms after adjusting for perceived stress. A better understanding of these two different pathways would be useful to guide the selection of intervention strategies that primarily address stress management or study environment-related factors.

Very little research has been conducted on mental health status among students in former socialist countries. Therefore, we assessed depressive symptoms, perceived stress, and perceived burdens related to being a student comparatively among university students from Germany, Poland, and Bulgaria. The specific aims were to: 1) investigate the association between perceived burdens and depressive symptoms, controlling for socio-economic characteristics and academic performance at the university, but not adjusting for stress (this is equivalent to the assumption that perceived stress is a mediating variable only and that the impact of stress on depressive symptoms is fully explained by perceived burdens and other variables in the model); 2) assess the effect of perceived burdens on depressive symptoms, controlling for stress (this is equivalent to investigating the effects of perceived burdens which are not mediated by stress); 3) assess the association between the variables used for adjustment in the above models and depressive symptoms.

## Methods

### Survey sample and questionnaire

The data was collected as part of the Cross-National Student Health Study (CNSHS), a collaboration among several European universities conducting student health surveys [14]. This analysis is based on surveys administered in 2005 at three universities: University of Bielefeld (Germany), Catholic University of Lublin (Poland), and Sofia University (Bulgaria). In further text we will use the country names to denote the respective samples. At each site, the first semester lectures/seminars were selected so as to obtain similarity between the samples: one third of the sample from natural sciences, one third from social sciences and languages, and one third from law and economy. Students were asked towards the end of lectures to complete the surveys. Student participation in the study was voluntary and anonymous; students were informed that by completing the questionnaire, they agree to participate in the study; and no incentives were provided. The participants completed a self-administered questionnaire covering a range of health issues, including mental health. The questionnaire was initially compiled in German language and was subsequently translated into Polish and Bulgarian employing two independent translators for each language. For both translations, cases of disagreement were resolved by the appropriate investigators who were native speakers in Polish (RTM) and Bulgarian (VN) and had expertise in survey research. The response rate was 85% in the German sample and above 95% in both the Polish and Bulgarian samples.

### Measures

*Depressive symptoms *were measured using the a modified version of Beck Depression Inventory (M-BDI), available in German language [15,16]. The Beck Depression Inventory (BDI) has been used since the early 1960s to identify and assess depressive symptoms and has an established validity and reliability [17]. The original BDI consists of 21 symptoms measured with four statements each, but subsequently, shorter versions have been developed [18]. The modification used in this analysis replaced each symptom's four statements with a single statement that used a 6-point Likert scale ranging from '0 = Never' to '6 = Almost Always'; and excluded the symptom of weight loss (thought to be not specific enough). The M-BDI items assess the frequency of depressive symptoms such as hopelessness and irritability, cognitions such as guilt or feelings of being punished, as well as physical symptoms such as fatigue, loss of appetite, and lack of interest in sex. The German language M-BDI, as all other versions of the BDI, computes a single score for individual respondents by summing their responses for all items of the scale. The construct and criterion validity and measurement equivalence of the M-BDI, as compared to the original BDI, have been previously demonstrated in a German general population sample and selected subsamples [15]. A cut-off score of ≥ 35 has been provided for screening for clinically relevant depression. This cut-off point had the best balance of sensitivity (92%) and specificity (91%) in screening for major depression, and corresponded to the 85^th ^percentile of a representative sample of the German population [19].

*Perceived stress *was assessed using the 14-item Perceived Stress Scale (PSS) [20], which measures the degree to which a respondent appraises situations his or her life as stressful. Items were designed to gauge how unpredictable, uncontrollable, and overloaded respondents find their lives. These 14 items used a 4-point Likert scale response format, ranging from '0 = Never' to '4 = Very Often.' Scores for individual respondents were obtained by averaging their responses to all the items of the scale. The internal reliability (Cronbach's alpha) of the scale in a probability sample of the U.S. population was 0.78 [21]. Predictive and discriminant validities of the scale have been demonstrated in several studies that relate the measured concept of stress to health outcomes [21,22]. Bulgarian and Polish translations of the PSS were performed for the purpose of this study.

*Perceived burdens *were measured by a scale that was developed for the purpose of this study based on responses to a shorter scale used in previous rounds of CNSHS. The scale assessed burdens associated with course work and exams, relationships to peers and parents, isolation, and expectations regarding the future. Items were introduced with the question: "To what extent do you feel burdened in the following areas?" In order to combine the items into appropriate subscales, an exploratory factor analysis using Varimax rotation with a Kaiser Criterion for factor extraction was performed separately for each of the three countries. After the factor structure was found to be very similar, the data from the three different countries were merged. The internal reliability of the subscales was assessed by Cronbach's alpha. Table 1 depicts the final composition, number of items, and internal reliabilities of the 4 subscales included in this analysis. Subscale scores were obtained by averaging responses of corresponding items.

**Table 1:** Measures used in this study and their internal reliability

No of items	Description and Response Scales	Cronbach's alpha		
		
		Germany	Poland	Bulgaria
20	**Depressive Symptoms: **Modified Beck Depression Index (6 point Likert scale: never – nearly always)	0.90	0.92	0.87
14	**Perceived Stress: **Cohen's perceived stress scale (5 point Likert scale: never – very often)	0.85	0.81	0.80
5	**Perceived Burdens**^+ ^– subscale: "Isolation" (Problems with fellow students; Isolation at the university; Isolation in general; Anonymity at university; Problems with friends)	0.77	0.75	0.81
2	**Perceived Burdens**^+ ^– subscale "Relationships" (Relationship with significant other; Sexuality)	0.84	0.79	0.78
4	**Perceived Burdens**^+ ^– subscale "Course work" (Studies in general; Exams; Assignments; Presentations)	0.79	0.83	0.77
2	**Perceived Burdens**^+ ^– subscale "Future" (Bad job prospects; Lack of practical relevance of studies)	0.46	0.74	0.63

^+^To what extent do you feel burdened in the following areas: (6 point Likert scale: Not at all – Very much)

*Other variables used in the analysis were *gender of the respondent; having a partner (boy-/girlfriend or spouse); respondent's subjective economic situation (measured by a single item: "How sufficient is your income?" on a 4-point response scale from 'Totally sufficient' to 'Not sufficient at all'); and performance at the university (measured by a single item: "How would you rate your performance at the university in comparison to others?" on a 5-point response scale from 'Much Better' to 'Much Worse,' subsequently collapsed into three categories for analysis: 'Worse' (much worse and worse), 'Average,' and 'Better' (much better and better)).

### Statistical analysis

For descriptive analysis, we tabulated frequencies and means. We used ANOVA and t-test for bivariate analysis. The correlations between the perceived burden subscales and PSSs ranged from 0.24 to 0.36. Based on Burnand, et al[23], we considered r < 0.30 to indicate negligible associations and 0.30 < r < 0.45 to indicate moderate correlations. We used two linear regression models to compare the total and stress independent effects of perceived burdens on depressive symptoms. Both models included nine independent variables and used the depression score as the outcome. Model 2 evaluated the change from total to stress independent effects by including a perceived stress score. We used the partial eta-square to evaluate the strength of association between the different variables and depressive symptoms. The partial eta-square is the amount of the effect and error variance attributable to the given variable, and as such it allows for the comparison of the effect size of different variables, independent of any other variables that are included in the model [24,25]. In model 2, which included perceived stress, all two-way interactions were investigated jointly. We applied backwards elimination based on the Wald test to exclude terms that were not significant at the ≤ 0.05 level. All statistical analyses were performed with SPSS^® ^12.

## Results

### Description of the sample

Across the three countries, the majority of students were females, with higher fractions of female students in both Slavic countries (Bulgaria and Poland) (Table 2). Bulgarian students were the youngest, followed by students in Poland and in Germany. Because of the small variability of age within the countries and the higher variability between the countries, no adjustments for age were performed in the subsequent analyses. Parental education was similar in Germany (17% of parents had a university education) and Poland (14%), but as much as 50% of the surveyed students in Bulgaria reported that both their parents had a university education. Since the participating universities had different academic structures and faculties, there were also minor differences in the participating students with respect to their area of study.

**Table 2:** Characteristics of the sample (%, unless indicated)

Variables	**Bielefeld, Germany **N = 803	**Lublin, Poland **N = 591	**Sofia, Bulgaria **N = 709
**Gender**			
Female	57.7	71.3	68.3
Male	42.3	28.7	31.7
**Age category**			
<20 years	1.8	22.9	55.2
20–22 years	72.0	74.9	42.5
>22 years	26.1	2.4	2.3
**Having a partner**			
Yes	55.6	35.5	42.3
No	44.4	64.5	57.7
**Parental education**			
Both university	17.8	14.1	49.9
Other	82.2	85.9	50.1
**Field of study**			
Health subjects	7.3	-	-
Basic science and engineering	27.1	17.3	26.5
Social science, art, teaching, languages	47.6	51.7	54.1
Business and law	17.8	19.3	19.4
Other	0.3	11.7	-
**Perceived burdens related to studying**			

	Mean [range] (SD)	Mean [range] (SD)	Mean [range] (SD)

Course work^+^	4.19 [1–6] (1.12)	4.20 [1–6] (1.19)	3.83 [1–6] (1.24)
Future^+^	3.16 [1–6] (1.18)	3.25 [1–6] (1.26)	3.26 [1–6] (1.32)
Relationships^+^	2.06 [1–6] (1.33)	2.06 [1–6] (1.37)	2.28 [1–6] (1.28)
Isolation^+^	1.90 [1–6] (0.81)	2.07 [1–5.2] (0.86)	1.88 [1–5.4] (0.91)
**Perceived stress**	1.73 [4–51] (0.57)	1.99 [10–52] (0.57)	1.83 [5–51] (0.56)

^+ ^To what extent do you feel burdened in the following areas: (6 points: 1 = 'Not at all' to 6 = 'Very much'); * ANOVA for comparison between countries

Students were more burdened by course work, taking exams, and completing assignments (subscale "Course work") and by bad job prospects and lack of practical relevance of their studies (subscale "Future") than by relationship problems (subscale "Relationships") or by feelings of being isolated (subscale "Isolation") (Table 2, bottom section). The overall pattern of perceived burdens was similar for all three countries. Perceived stress was highest in Poland, followed by Bulgaria and Germany.

In general, the M-BDI scores were relatively high, with 23% of German students, 34% of Polish students, and 39% of Bulgarian students exhibiting scores ≥ 35. The higher values among Bulgarian students resulted from the whole distribution being shifted to the right. Whereas among Polish students there was larger heterogeneity, with a hint of bimodality in the distribution and a second peak at an M-BDI score of about 40 (not shown). The findings regarding the shape of the distribution were similar for both genders.

### Bivariate analysis of associations between different factors and depressive symptoms

Most of the categorical variables were significantly associated with the M-BDI score (Table 3). Mean scores for M-BDI were similar for the Slavic countries and considerably lower for Germany. Male gender, having a partner, and sufficient income were associated with lower depression scores, whereas low performance at the university compared to other students was associated with considerably higher scores. Among continuous characteristics, all perceived burdens as well as perceived stress were correlated with the depression score.

**Table 3:** Modified Beck Depression Index (M-BDI) for different subgroups and correlations between M-BDI and continuous variables

Categorical variables	Mean M-BDI (SD)	p-value*
University		<0.001
Bielefeld, Germany	26.87 (15.25)	
Lublin, Poland	31.87 (16.91)	
Sofia, Bulgaria	32.50 (14.97)	
Gender		<0.001
Female	31.78 (16.20)	
Male	27.29 (14.53)	
Having a partner		<0.001
Yes	27.93 (14.93)	
No	32.19 (16.31)	
Sufficiency of income		<0.001
Totally sufficient	26.32 (14.83)	
Sufficient	28.60 (14.76)	
Not sufficient	31.14 (15.69)	
Not sufficient at all	36.32 (17.35)	
Performance at the university		<0.001
Worse	37.48 (16.93)	
Average	28.09 (15.10)	
Better	28.94 (14.64)	
Continuous variables	Correlation coefficient (Pearson r)	p-value^+^
Perceived burdens: Isolation^#^	0.438	<0.001
Perceived burdens: Relationships^#^	0.290	<0.001
Perceived burdens: Course work^#^	0.275	<0.001
Perceived burdens: Future^#^	0.290	<0.001
Perceived stress^§^	0.702	<0.001

* ANOVA for comparison between categories; ^+ ^t-test for Pearson coefficient = 0; ^# ^To what extent do you feel burdened in the following areas: (6 point Likert scale: 'Not at all' – 'Very much'); ^§ ^Cohen's perceived stress scale (5 point Likert scale: 0 = 'Never' to 4 = 'Very often')

### Role of perceived stress in the association between perceived burdens and depressive symptoms

In model 1 (Table 4), where the total effects of perceived burdens were investigated (including the effect of burdens mediated by stress), all the included variables were significantly associated with the depression score. However, when perceived stress was included as an additional independent variable (model 2, Table 4), the independent effects of most of the predictors were less pronounced. In particular there were two notable changes: gender and perceived burdens related to course work and exams (subscale: "Course work") no longer displayed independent effects on the depression score.

**Table 4:** Total and stress independent associations between different variables and the depression score (p-value obtained from F-test and partial eta^2 ^obtained from multiple analysis of covariance)

Variable	Model 1^a^ p-value/partial eta^2^	Model 2^b^ p-value/partial eta^2^
Full model	<0.001/0.333	<0.001/0.568
Perceived burdens: Course work	<0.001/0.035	<0.445/0.001
Perceived burdens: Isolation	<0.001/0.083	<0.001/0.047
Perceived burdens: Relationships	<0.001/0.022	<0.001/0.008
Perceived burdens: Future	<0.001/0.010	<0.001/0.009
Countries	<0.001/0.035	<0.001/0.027
Gender	<0.001/0.015	<0.157/0.001
Having a partner	0.001/0.007	0.023/0.003
Sufficiency of income	0.001/0.011	0.043/0.005
Performance at the university	<0.001/0.039	<0.001/0.017
Perceived stress	(not included in the model)	<0.001/0.351

^a ^assuming that stress is only an intermediate effect between other variables in the model and depression, thus estimating the total effect of perceived burdens etc.

^b ^including all predictors and perceived stress, thus estimating the effect of other variables controlling for stress

### Association of adjusting variables with depressive symptoms

In the next step of analysis, all possible two-way interactions in model 2 were investigated. Only the interaction between gender and perceived stress proved significant. Among the subscales of burdens only three remained significantly associated with the depression score (Table 5). Interestingly, the impact of perceived burdens, gender, performance at the university, and perceived stress on depressive symptoms was not different across the three countries (indicated by the lack of interaction between these variables and country). Apart from the interaction with stress, the same was also true for gender. Isolation, the burden subscale that was least experienced by students, had a much higher impact on depression scores than burden subscales related to personal relationships or future prospects. Also, in contrast to the bivariate analysis, which depicted similarly higher mean M-BDI scores for Poland and Bulgaria than for Germany, Poland and Germany now appeared more similar to one another with regard to depressive symptoms: students in both countries had lower levels of depressive symptoms than students in Bulgaria. A stepwise inclusion of variables, starting with country, showed that this change was introduced by adjusting for perceived stress. The mean perceived stress score was higher in Poland than in Bulgaria (and Germany). Since there was no difference in the effect of stress on depressive symptoms between countries, the adjustment for stress resulted in downwardly shifting Poland's depressive symptoms estimate toward the estimate for Germany. Of all the variables included in the analysis, perceived stress had the highest impact on depression score. The impact of perceived stress was higher among males than among females, however, females had, on average, higher stress scores than males (1.92 versus 1.70, p < 0.001 [26]). Better academic performance at the university was associated with lower depression scores, with the average academic performance group having the lowest M-BDI score.

**Table 5:** Change in M-BDI score according to different characteristics

Variable	Linear coefficient (95% CI)	p-value^+^
Perceived burdens: Isolation*	3.19 (2.51; 3.88)	<0.001
Perceived burdens: Relationships*	0.86 (0.45; 1.26)	<0.001
Perceived burdens: Future*	0.88 (0.45; 1.31)	<0.001
Country		
Germany vs. Bulgaria	-3.97 (-5.16; -2.79)	<0.001
Poland vs. Bulgaria	-3.70 (-5.05; -2.35)	<0.001
Gender		
Male vs. female	-6.06 (-9.52; -2.60)	0.001
Academic performance at the university		
Better vs. worse	-2.74 (-4.27; -1.20)	<0.001
Equal vs. worse	-3.88 (-5.21; -2.54)	<0.001
Perceived stress^# ^(female)	13.27 (11.69; 14.85)	<0.001
Perceived stress^# ^(male)	17.05 (14.60; 19.50)	<0.001

* per unit change, scale from 1 = 'Not at all' to 6 = 'Very much');

^+ ^Wald-test;

^# ^per unit change, scale from 0 = 'Never' to 4 = 'Very often'.

## Discussion

This study analysed depressive symptoms in student samples from three universities in three European countries and their association with perceived burdens related to being a student. We found a relatively high level of depressive symptoms as compared to a representative sample of the German population, where the 85^th ^percentile of the M-BDI for 20 to 30 year olds is 35 for the total sample, with a five point difference between genders [19]. Similarly, previous reports have shown high levels of depressive symptoms among college or university students [1-7]. Our bivariate finding of higher depression scores among students in Bulgaria and Poland as compared to those from Germany is consistent with two previous studies that reported substantially higher prevalences of depressive symptoms among student samples in Eastern Europe than in Western Europe [27]. However, the difference in M-BDI scores between Poland and Germany disappeared after adjusting for perceived stress, which was particularly high in Poland.

All four subscales of perceived burden were associated with depressive symptoms and, apart from burdens related to course work and exams (subscale "Course work"), the associations persisted after adjusting for perceived stress. Since perceived stress explained the association between the "Course work" burden subscale and depressive symptoms, it seems that stress fully mediated the effects of these burdens. On the contrary, perceived stress only partially acted as a mediator for the "Isolation" and "Relationships" subscales, and did not act as a mediator at all for the "Future" subscale (compare partial eta-square values from models 1 and 2 in Table 4). These findings partly support the distinction between total and mediated effects specified in our aims. While perceived burdens have some conceptual similarity with perceived stress, they have also independent association with depressive symptoms. This may be a result of the perceived stress scale measuring only selected aspects of stress or may be a result of a true conceptual distinction between perceived burdens and stress. Our findings on the extent to which the subscales' associations were mediated by perceived stress ("Course work" the most and "Future" the least) support the suggestion of a true conceptual distinction. Course work is most directly a stressor in students' lives, and the concept of perceived stress is mostly related to the present time, not to the future outlook. This supports the importance of stress management interventions for student health, but also indicates that interventions addressing perceived burdens would be useful.

Only few studies have examined perceived burdens related to being a student, such as grades and competition, career and future success, too many demands and deadlines, financial and health-related burdens [28], and their impact on mental health [29]. Our findings suggest that more students felt burdened by their course work and exams and an uncertain outlook for the future than by problems with relationships or being isolated, with little differences between countries. When present, however, the feeling of being isolated, had the strongest impact on depressive symptoms; a fact which highlights the importance of social support and the sense of belonging to a group while being a student.

Similarly, economic situation (measured by sufficiency of income) mainly had an indirect effect on depressive symptoms (mediated over perceived stress). After controlling for perceived stress, sufficiency of income was no longer significantly associated with depressive symptoms. These findings underline findings from previous studies that report a strong association between stress and depressive symptoms [13].

Low perceived academic performance at the university was associated with higher depression scores. Depression scores were lower in the group rating their academic performance highest than in the group with lowest performance, but the lowest depression scores were found among students considering themselves average. This suggests a stronger disposition or tendency towards depression or more pressures in the group with the best academic performance. Low performance may be both a cause and a consequence of depressive symptoms. Other studies have found that students with higher depressive symptoms engage more often in social comparison processes and react more negatively to upward comparisons [30].

Perceived stress had the strongest association with the depression score in both genders. But whereas females had higher perceived stress scores on average, the impact of perceived stress on depressive symptoms was stronger for males. Gender differences in depressive symptoms increase from preadolescence to young adulthood, evolving around the age of 14 years [31] and reaching a maximum t about 16–18 years of age [32]. During this same age span, stress also increases in adolescent girls [33]. Avison and McAlpine [34] showed that the higher level of depression in female high school students was explained by higher levels of stress. In our analysis, the female gender continued to be associated with higher M-BDI scores in the bivariate analysis and after adjusting for other variables in the multivariate analysis, until perceived stress was included in the model (which would agree with the above finding). When perceived stress was included in the model only as the main effect, the gender difference disappeared. However, the gender difference reappeared with the interaction between gender and stress score included in the model. Whereas male students had lower depressive symptoms for low levels of stress as compared to females, their depressive symptoms increased more strongly with stress. This would suggest an additional gender difference with respect to coping with stress and its impact on depressive symptoms, which was not examined previously.

In samples that include older adults, higher depression scores are usually associated with a lack of an intimate or marital relationship [35]. We did find significantly lower depression scores among students who had an intimate partner as compared to those who did not have a partner in the bivariate analysis. However, this association was weak and disappeared after adjusting for perceived stress. Due to the likely rich network of social interactions among students and in university life, we would not expect having a partner to make a strong impact on depression scores Though, since perceived stress does (albeit weakly) mediate the effect of having a partner on depression scores, there is an indication that some level of stress could be associated with not having a partner, even in our relatively young sample.

### Limitations

The sample employed in this study was restricted to first-year students from only one university per country. Thus caution should be exercised in generalizing these findings to all students. The differences we found between the countries might only reflect differences between the three universities that participated in the study. However, we are not aware of any reason to believe that students at these three universities are different from students at other universities in these countries. At each survey site, we achieved a high response rate, preventing selection bias within the investigated populations. We used a relatively recent modification of BDI with the one drawback of limited information (restricted to German samples) on the validity and reliability of the M-BDI [16,19]. The study only measured depressive symptoms during the past few days. No information was collected on events within or outside the university setting, which could have an affect on depressive symptoms. At all three universities, the survey was conducted during the second half of the summer term, when end of the term exams were not too distant. However, since the timing of the survey was similar at all three universities, this should not have biased our comparisons. The instrument used to measure perceived burdens was not established prior to this study and, apart of the analysis of internal reliability, no further investigations were performed. Given the cross-sectional character of our study, all investigated relationships were associations and no inference about causal association can be made. In particular, depressive symptoms could have also manifested in higher scores on perceived burdens and stress scales.

## Conclusion

In summary, students displayed levels of depressive symptoms that were relatively high as compared to the general population in Germany, with even higher levels in the two Eastern European countries. Consistent with other studies, perceived stress was strongly associated with higher depression scores. Previous research has also indicated a difference between Western and Eastern European countries, but our study showed that the difference in depression scores between Germany (Western) and Poland (Eastern) disappeared after adjusting for perceived stress. In addition, perceived burdens were also associated with depressive symptoms. Depending on the subscale, the effects were totally ("Course work"), partially ("Isolation" and "Relationships"), or not ("Future") mediated by perceived stress. These findings were similar for all three countries. While the strong association between perceived stress and depressive symptoms suggests the need for interventions that improve stress management, perceived burdens should also be addressed.

## Competing interests

The authors declare that they have no competing interests.

## Authors' contributions

RTM contributed to the research question, performed the analysis and wrote a draft of the manuscript. AEM developed the research question and wrote the final version of the manuscript. VN, SM, WEA contributed to writing of the manuscript.
